# Analysis of Medical Error Contributing to Missed Acute Myeloid Leukemia Diagnosis

**DOI:** 10.7759/cureus.4449

**Published:** 2019-04-13

**Authors:** Chelsea Angel, Clifford D Packer

**Affiliations:** 1 Internal Medicine, Case Western Reserve University School of Medicine, Cleveland, USA

**Keywords:** medical error, oncology, hematology, pancytopenia, internal medicine

## Abstract

Hospitals and physicians attempt to minimize medical error by putting systems checks and balances in place at multiple levels. The effectiveness of these hospital-specific strategies to thwart error is called into question, as medical error remains a leading cause of death in the United States. This case report outlines the course of a 62-year-old man with a history of non-small cell lung cancer and right tongue squamous cell carcinoma, who had been admitted to an outside hospital for possible pneumonia. On initial presentation, the patient was pancytopenic with an absolute neutrophil count of 598. As his counts continued to downtrend and his conditioned worsened, oncology saw the patient and attributed the pancytopenia to "transient myelosuppression from pneumonia”. This statement impacted the trajectory of the patient’s care, delaying his ultimate diagnosis and treatment for acute myeloid leukemia. This case emphasizes the power of framing and anchoring biases in medical decision making and the need to evolve practice models from the current method of closed-door inquiry towards a more inclusive system of error reporting and analysis.

## Introduction

The British Medical Journal defines medical error as, “an unintended act (either of omission or commission) or one that does not achieve its intended outcome, the failure of a planned action to be completed as intended (an error of execution), the use of a wrong plan to achieve an aim (an error of planning), or a deviation from the process of care that may or may not cause harm to the patient” [1]. Hospitals and physicians attempt to combat these unfortunate events by putting systems checks and balances in place to act as barriers against error at multiple levels, known as The Swiss Cheese Model [2, 3]. There are times, however, when the holes may align and error occurs leading to, what some analysts estimate, the third leading cause of death in the USA [1]. Acute myeloid leukemia (AML) is a rapidly progressing cancer that, without treatment, can lead to death within days to weeks of onset [4]. The initial presentation is often non-specific, with constitutional symptoms such as weakness and fatigue, or other symptoms related to complications of pancytopenia [4]. Therefore, high index of suspicion and rapid identification and diagnosis of AML is paramount to optimize patient outcomes and decrease mortality. In this case report, we analyze a case of a missed AML diagnosis in a patient with shortness of breath and pancytopenia, to assess where the system failed and what safeguards could have prevented this diagnostic error.

## Case presentation

The patient is a 62-year-old man with a history of non-small cell lung cancer status post chemoradiation, chronic obstructive pulmonary disease (COPD), right tongue squamous cell carcinoma status post right partial glossectomy and neck dissection followed by chemoradiation, who had been admitted to an outside hospital for possible pneumonia. Upon admission, he was found to have pancytopenia with white blood cell (WBC) 2600, hematocrit 36.6%, platelet count 62,000, and absolute neutrophil count (ANC) 598. As the ANC continued to downtrend, oncology saw the patient and commented that the "pancytopenia is likely from transient myelosuppression from pneumonia”. He was released from the hospital five days later with antibiotics.

Nine days later, he saw his primary care physician (PCP) for hospital follow-up who wrote:

"His white count went as low as 1600 on his recent hospitalization, but had increased bands and metamyelocytes and was thought to have some transient marrow suppression secondary to infection or medications…They also advised him to follow up with outside infectious disease and hematology, although I see little need for this."

Routine blood work a few days later revealed worsening pancytopenia. He was advised to go to the emergency room and was admitted to the hospital. The day following admission, flow cytometry was sent due to high suspicion of leukemia. A week later, almost three and a half weeks after this initial presentation of pancytopenia, with ANCs reaching as low as 280, results confirmed diagnosis of AML and chemotherapy was initiated. The patient began to decline rapidly and was transferred to the medical intensive care unit (MICU). There, he experienced respiratory failure that required intubation. A few days later, the decision to hold chemotherapy was made. The patient became anuric and ultimately developed vancomycin-resistant Enterococcus (VRE) bacteremia. After discussing his prognosis with his wife, the decision was made to extubate and treat with comfort care. The patient died less than a month later.

## Discussion

For this patient, the “holes in the Swiss Cheese” aligned to miss the diagnosis of AML. In this case, the physicians’ approach to this patient’s pancytopenia was likely impacted by framing and anchoring biases [5-7]. Framing and anchoring are cognitive biases that have been well studied since their initial elucidation in 1974 [8]. Framing bias describes the phenomenon of reacting to information differently based on how it is presented, whereas anchoring bias is the predilection to fixate on the initial diagnostic impression, with all future data analyzed in reference to that “anchor” [5, 8]. Since the patient had history of COPD exacerbation and pneumonia, his physicians were inclined to frame the new pancytopenia in the context of his respiratory infection. Furthermore, once the oncologist confirmed this impression by stating the patient had “transient myelosuppression from pneumonia,” all subsequent progress notes anchored around that statement. While it is true that infection can cause pancytopenia, the five leading infectious causes of pancytopenia are AIDS, septicemia, enteric fever, tuberculosis, and viral hepatitis [9]; our patient presented initially with a community-acquired pneumonia without evidence of septicemia. The presence of metamyelocytes on the peripheral smear and the prior history of two malignancies treated with chemotherapy and radiation should have raised concern for a new hematologic malignancy. Currently, approximately 65-70% of adult AML patients <60 years old and 25-40% of patients who are >60 years old reach a complete remission with treatment [4, 10]. It is possible that early detection, diagnosis, and treatment of our patient’s AML could have prevented his rapid deterioration and ultimate death.

## Conclusions

Physicians’ susceptibility to framing and anchoring biases can delay diagnosis and adversely affect medical decision making. To avoid these biases, it is important to reappraise the working diagnosis as new evidence becomes available and accept diagnostic uncertainty. Ideally, discussion of uncertainty and reappraisal of the working diagnosis should become a part of the daily routine, both on rounds and with every patient hand-off or transfer. In current practice, however, patient deaths attributed to medical error tend to be discussed in a limited capacity, with individual hospital systems assuming the responsibility of addressing perceived “holes” in their defenses as they arise. One could argue that the medical community would benefit from pooled reports and samples of error to be publicized and discussed openly, as is the usual practice in the wider scientific community. A universal system for reporting and analysis of medical errors might help to identify and ultimately prevent the biases and system failures that lead to serious medical error.
